# Anti-HIV and immune modulating activities of IND02

**DOI:** 10.1186/1742-4690-9-S2-P220

**Published:** 2012-09-13

**Authors:** ME Biedma, B Connell, S Schmidt, H Lortat-Jacob, C Moog, E Prakash

**Affiliations:** 1 Instituté de Virologie, INSERM U748, Strasbourg, France; 2 Institut de Biologie Structurale, Grenoble, France; 3 Indus biotech Pvt Ltd, Pune, India

## Background

A vaccine that prevents HIV infection should not only induce functional inhibitory, neutralizing antibodies, but also promote Fc-mediated inhibitory antibodies displaying ADCC or phagocytosis. The aim of this study is to analyze the effect of IND02, a cinnamon derived procyanidin polymer on the interaction of HIV-1 gp120 with its co-receptors as well as its adjuvant like activity along with HIV-specific antibodies.

## Methods

The ability of IND02 to interact with the HIV envelope glycoprotein gp120 was analyzed by studying binding of IND02 to gp120 envelopes by Biacore. The expressions of FcγRs were evaluated on macrophages and NK primary cells incubated with IND02 by flow cytometry. The inhibitory activity of IND02 was assessed on PBMC using TZM-bl assay for neutralization, Fc-mediated inhibitory activity on macrophages and Antibody Dependant Cellular Cytotoxicity in presence or absence of anti-HIV antibodies.

## Results

Binding of IND-02 to gp120 envelopes was dose-dependent within the μM range and was capable to inhibit gp120-CD4 interaction. A moderate decrease in the expression of FcγR I and III was observed on macrophages treated with IND02, while FcγR II expression was unaffected. FcγRIII expression on NK cells was not modified. IND02 demonstrated low inhibitory activity on TZMbL and PBMC, but was able to efficiently inhibit HIV infection on macrophages. Moreover IND02 demonstrated synergistic effect when combined with monoclonal inhibitory antibodies. An enhanced ADCC was detected in presence of IND02 and anti-HIV specific antibodies.

## Conclusion

IND02 represents an interesting class of botanical molecule that binds to HIV-1 envelope protein, including the co receptor binding site, impairing HIV interaction with co-receptors on target cells. This interaction could explain the decreased HIV replication observed after IND02 treatment. Besides, an augmentation in the activity of HIV-specific antibodies, involving ADCC and Fc-mediated phagocytosis, was observed in presence of IND02 supporting additional adjuvant mechanisms that could enhance immune responses against HIV.

